# The Semantic Automated Discovery and Integration (SADI) Web service Design-Pattern, API and Reference Implementation

**DOI:** 10.1186/2041-1480-2-8

**Published:** 2011-10-24

**Authors:** Mark D Wilkinson, Benjamin Vandervalk, Luke McCarthy

**Affiliations:** 1 Department of Medical Genetics, Heart + Lung Institute at St. Paul's Hospital, University of British Columbia, Vancouver, BC, Canada

## Abstract

**Background:**

The complexity and inter-related nature of biological data poses a difficult challenge for data and tool integration. There has been a proliferation of interoperability standards and projects over the past decade, none of which has been widely adopted by the bioinformatics community. Recent attempts have focused on the use of semantics to assist integration, and Semantic Web technologies are being welcomed by this community.

**Description:**

SADI - Semantic Automated Discovery and Integration - is a lightweight set of fully standards-compliant Semantic Web service design patterns that simplify the publication of services of the type commonly found in bioinformatics and other scientific domains. Using Semantic Web technologies at every level of the Web services "stack", SADI services consume and produce instances of OWL Classes following a small number of very straightforward best-practices. In addition, we provide codebases that support these best-practices, and plug-in tools to popular developer and client software that dramatically simplify deployment of services by providers, and the discovery and utilization of those services by their consumers.

**Conclusions:**

SADI Services are fully compliant with, and utilize only foundational Web standards; are simple to create and maintain for service providers; and can be discovered and utilized in a very intuitive way by biologist end-users. In addition, the SADI design patterns significantly improve the ability of software to automatically discover appropriate services based on user-needs, and automatically chain these into complex analytical workflows. We show that, when resources are exposed through SADI, data compliant with a given ontological model can be automatically gathered, or generated, from these distributed, non-coordinating resources - a behaviour we have not observed in any other Semantic system. Finally, we show that, using SADI, data dynamically generated from Web services can be explored in a manner very similar to data housed in static triple-stores, thus facilitating the intersection of Web services and Semantic Web technologies.

## Background

Two Web technologies - Web services and the Semantic Web - hold the promise to achieve integration and interoperability among the currently disparate bioinformatics resources on the Web; however, this promise is not being widely achieved in practice. The causes of failure are varied, but often relate to the fundamental differences between the Web service and Semantic Web technologies themselves, and the widely varying approaches taken by different projects who have attempted to superimpose one technology over the other.

Archetypal Web services adopt a request/response model that utilizes HTTP POST as the transport layer, and a technology called Simple Object Access Protocol (SOAP) to surround the input/output messages with informative metadata. The functions made available by the Web service are described via a machine-readable specification called Web Services Description Language (WSDL)[1], which in turn utilizes XML Schema to describe the syntactic structure of the each function's input and output messages. If the "meaning" of the syntactic XML elements of an output message, and a desired subsequent input message, are known (or can be inferred) it is possible to chain Web services together into workflows. However, because of the lack of shared semantics regarding the meaning of elements in an XML Schema, workflow design is most commonly done manually in an editing environment such as Taverna [2], and the promise of automated Web service interoperability and workflow construction is only truly successful within well-defined, often project-specific situations.

Defining these shared semantics is one of the aims of the emergent Semantic Web initiative [3]. The Semantic Web can be thought of as a directed-graph in which the nodes are anything that can be named (a concept, a document, a person) and the labelled edges are meaningful properties that describe the relationships between the nodes. Resource Description Framework (RDF) [4] is a way of encoding these nodes and labelled edges such that they can be explored and traversed by machines, and most of the data on the Semantic Web is currently stored in RDF documents made available by HTTP GET, or in "triple-stores", which are the RDF equivalent of relational databases. All nodes and properties in RDF datasets are referenced by globally unique identifiers (Uniform Resource Identifiers (URIs)), and thus the encoding provided by RDF is precise, unambiguous, and ideally suited for automated processing by software. Moreover, it is possible to use logical reasoning to derive new facts which are not explicitly stated in the data. Description logics (DL) are typically employed for this purpose due to their improved computational characteristics in comparison to first order logic, and OWL [5] is the family of description logics that has been developed for use with the Semantic Web.

Here we describe our attempt to merge these two technologies in a way that directly addresses the needs and behaviours of a specific end-user community, namely bioinformaticians, who have strong resource and data interoperability requirements. SADI - Semantic Automated Discovery and Integration - is a novel Semantic Web service design-pattern, and supporting codebase together with a reference implementation, that utilizes Semantic Web standards at all levels of the Web services "stack", including discovery, messaging, and service description. Following Carole Goble's advice that "any integration technology should only be as heavy as it needs to be, and no heavier" [6] SADI does not propose any new technologies, standards, messaging formats or structures, metadata structures, result codes, or unusual Web behaviours. SADI simply comprises a set of standards-compliant conventions and suggested best-practices for data representation and exchange between Semantic Web services that fully utilizes Semantic Web technologies to achieve the integrative behaviours required by our target community.

For service providers, adopting SADI has many advantages:

• The SADI design patterns are supported by an accompanying codebase and plug-in tools that almost completely automate the provision of resources as a Semantic Web service, leaving the provider to focus entirely on their business-logic.

• The simplicity of the approach also means that there are few places a provider can go wrong outside of the data model and their own business logic.

• Many of the decisions that need to be made when deploying Web services (of any kind) have been made in these design patterns, and have been made specifically to enhance service discoverability and interoperability. This simplifies the planning process for providers, by reducing the number of 'arbitrary' decisions they need to make.

• SADI services are easy to integrate with one another, greatly facilitating the construction of analytical pipelines, and therefore enhancing the usability of these services by the target end-users. This is made even simpler by the availability of SADI plug-ins to popular workflow clients such as Taverna [7] and data exploration environments such as the Knowledge Explorer [8] that dramatically simplify service discovery and pipeline creation.

• SADI is cluster/cloud-ready, and the specification was specifically designed to support multiplexed messages. This allows service providers to distribute incoming requests over their computational resources without any requirement for request/response tracking; responses from each processor may simply be concatenated regardless of order. Moreover, we utilize standard RDF-based approaches to avoid passing large datasets through workflows, and rather allow clients and providers to pass-by-reference.

• SADI enforces other best-practices in Web development (e.g. that all URIs must resolve), thus helping providers generate robust, error-free systems, and tools are available to regularly evaluate and validate service functionality. This results in high up-time, automated failure alerts, and therefore a higher quality of service for end-users.

• Service providers do not need to "buy-in" to any particular ontology, specialized protocol or message scaffold. SADI is agnostic to which ontologies are used to describe its messages, reducing the "friction" of bringing the technology into a new environment. SADI simply requires that providers utilize the Semantic Web standards of RDF and OWL for their data representation and modelling, under whichever ontological framework they wish.

• SADI is not in conflict with any existing network security software or protection model. It concerns itself only with how services behave, and simply passes plain-text messages via the standard HTTP Protocol.

Here we will first describe the SADI approach to Web service provision (an extension of the description here [9]). We will then briefly describe two implementations that show how the conventions and practices defined by SADI enable novel data discovery, interoperability, and integrative behaviors that we believe closely mirror the needs and expectations of our specific end-user community. Finally, we will engage in an extensive discussion of how SADI compares to peer technologies and other Semantic Web service projects.

The next section of this manuscript examines SADI iteratively, with increasing levels of detail at each iteration, such that the simplicity of the approach is made apparent before discussing the finer points of how SADI's integrative behaviours are achieved.

## Construction and Content

### Introduction - Hello World

**Figure 1:**
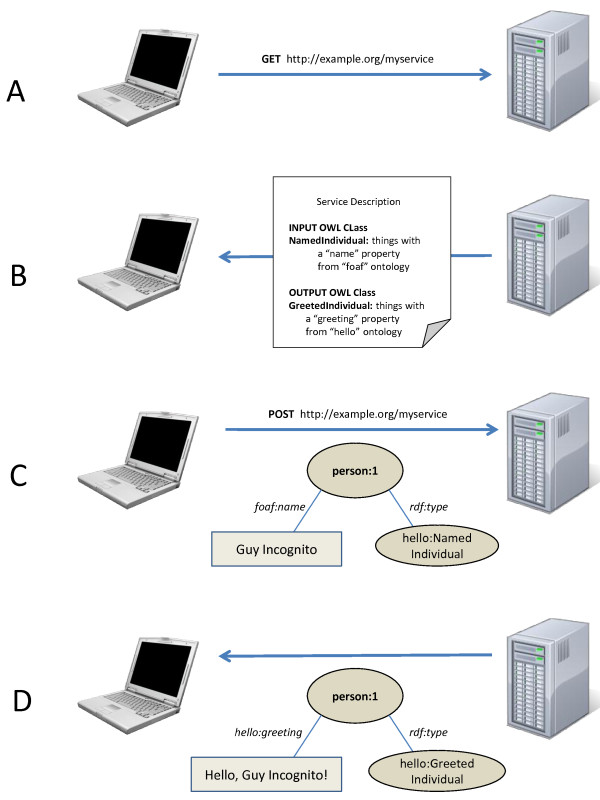
**The most basic SADI service transaction**. In (A) the client calls HTTP GET on the service endpoint. This results in the retrieval of a service interface document (B) containing references to OWL classes (defined anywhere on the Web) that describe the input and output datatypes of that service. The client finds RDF data matching the service's input OWL class (based on the property restrictions of that class) and passes that data to the service endpoint using simple HTTP POST (C). The service strips the properties from the input RDF node, uses that information to execute its analysis, and adds the results as new properties of the input node before returning it to the client as appropriately typed output (D).

Figure 1 shows a simple, synchronous interaction with a SADI service. A client calls HTTP GET on the service endpoint in order to retrieve the service interface document (Figure 1A). This document contains two OWL Class definitions, one describing the properties that must be carried by input data, and the other describing the properties that will be carried by the output data (Figure 1B). The client utilizes the input OWL Class to validate their desired input data (through logical reasoning), then passes that data *verbatim *to the service endpoint through a standard HTTP POST (Figure 1C). The service processes the input, and returns RDF data carrying the properties described in its output OWL Class; these represent the output of the service's processing (Figure 1D). While this appears to be (and is) an extremely straightforward and standard Web transaction, it embodies several simple constraints that make Web services modeled in this way highly discoverable and interoperable.

### SADI Approach to Semantic Web service modeling

Before describing SADI in detail, it is important to emphasise what SADI is *not*. SADI is not a protocol (e.g. not a replacement for SOAP), is not a registry (e.g. not a replacement for UDDI[10]), is not a data-typing system or ontology (unlike BioMoby[11]), and is not a service metadata or annotation schema (e.g. not a replacement for OWL-S[12], SAWSDL[13], or Feta[14]). SADI simply consists of a number of recommendations for how services themselves should be implemented and described in order to achieve a set of useful, interoperable behaviors that can be leveraged by existing Web service standards. As such, SADI is extremely lightweight compared to many other approaches to Semantic service provision. It consists of two key best-practices:

1. All service input and output data are RDF instances (i.e. owl:Individual's) of OWL classes

2. The URI of the output instance is the same as the URI of the input instance.

Best-practice #1enables sophisticated and flexible matchmaking between in-hand data and tools that can operate on that data, and does so using an increasingly widely-used data representation format - RDF. Best-practice #2 effectively standardizes the behaviour of all services by making them all "annotators", where the input becomes decorated by additional information before being returned to the client. This latter constraint has several very useful consequences, perhaps the most important being that the semantics of the underlying service functionality becomes extremely transparent. This greatly facilitates automated service discovery and pipelining as will be discussed and demonstrated below.

In the following sections, we will first describe the fundamental recommendations that apply to all SADI-compliant services, and will then describe extensions to the core recommendations that apply to, for example, asynchronous services or services that require additional parameters to alter service functionality.

### The Base SADI Specification

The core recommendations/requirements for a SADI compliant Web Service are listed in Table 1. Examining each of these recommendations in more detail will clarify more precisely what the service behaviour should be, why the decision was made and/or what benefit is gained by following the recommendation.

**Table 1:** Core Recommendations of SADI

1	SADI Web services are stateless and atomic.
2	SADI service endpoints respond to HTTP GET by returning the interface definition of the service.

3	Service interfaces (i.e., inputs and outputs) are defined in terms of OWL-DL classes; the property restrictions on these OWL classes define what specific data elements are required by the service and what data will be provided by the service, respectively.

4	SADI services consume and produce data in RDF format.

5	SADI services are invoked through plain HTTP POST of RDF data to the service endpoint.

6	Input RDF data - data that is compliant with (i.e. classifies into) the input OWL Class definition - is "decorated" or "annotated" by the service provider to include new properties until it fulfills the Class definition of the service's output OWL Class. Importantly, in so doing, the URI of the input OWL Class Instance is preserved and becomes the URI of the output OWL Class Instance.

### Explanation/Justification for Base Recommendations

#### SADI Web services are stateless and atomic

This decision is simply pragmatic, and describes the vast majority of services in the bioinformatics domain. Restricting the range of possible service behaviours to only those that are in-use simplifies the architecture. Services that cannot be modeled in a stateless manner - for example, simulation services - are not the immediate target of the SADI recommendations. That said, the flexibility of SADI's input and output data-typing should allow service providers considerable leeway in implementing services that behave in ways we had not anticipated; however, defining these behaviours is beyond the scope of the core SADI recommendations.

#### Service interface is retrieved by HTTP GET

It is useful to have a standard way of locating the service interface description for any given service. With WSDL-based services, locating these documents was only possible through *a priori *knowledge of the URL of the WSDL, or through querying a service registry. With SADI, we have standardized this such that the service endpoint itself responds to a GET by returning its service interface document (Figure 1A/B). Since (as described below) all SADI services function through HTTP POST, there is no barrier to restricting the use of GET in this way.

SADI does not define the format of the service interface document; however currently all SADI services return an RDF-XML instance (owl:Individual) of the serviceDescription Class from the myGrid/Moby service ontology [15]. This was chosen because the myGrid/Moby ontology has useful features for assisting with, for example, automated service monitoring, and moreover these annotations are compatible with the BioCatalogue[16] global registry of Web services. We are, however, actively monitoring alternatives, such as OWL-S, to determine if they become more widely accepted and/or more appropriate for the needs of SADI.

#### SADI services consume and produce RDF instances of OWL-DL Classes

Included in the service interface document are references to the OWL-DL classes that define the input and output data-types that the service will consume and produce. The ontologies defining those classes may exist anywhere on the Web, and may or may not be "owned by" the service provider; however, the URI of the input and output class *must *resolve, through HTTP GET, to an OWL document. SADI allows any provider to utilize classes from any OWL ontology within the definition of their own service interface.

The data consumed by a SADI service is an instance of the OWL-DL class that describes the input of the service (Figure 1C). Likewise, the output is an instance of the output class (Figure 1D). Both RDF-XML and RDF-N3 serializations are currently supported, and is indicated in the Content-type element of the HTTP header.

Since both the client and the service are operating on potentially very large RDF Graphs, it is important to indicate what URI(s) within that graph represent the "root" of the data instances. Here again we rely entirely on Semantic Web standards, requiring that the input instance must be classified according to the service provider's input class, and explicitly typed using the rdf:type predicate (See the "hello:NamedIndividual" node in Figure 1C). This serves to reduce the complexity of service provision by not requiring providers to reason over incoming data - an important consideration with respect to encouraging widespread adoption of SADI. Moreover, it allows services to be written in languages that do not have strong support for logical reasoners, such as Perl. When accepting incoming data, a provider simply extracts the URI from the input document that has the rdf:type property with a value equivalent to that service's input class. Client software can similarly expect that the service provider has added the rdf:type property to its root output data node (see the "hello:GreetedIndividual" node in Figure 1D), in accordance with its output class, and thus it is similarly straightforward for the client to identify output data elements within the returned graph.

#### Services are invoked by HTTP POST

SADI services are invoked by passing an RDF graph to the service end-point via HTTP POST (Figure 1C), and any tool that can execute an HTTP POST (e.g. Unix "curl") can be used to invoke a SADI service. Importantly, SADI uses a non-parameterized POST - i.e. does not use the HTTP FORM encoding. As such, all information required for service invocation must be present in the data itself, since the invocation happens via a single anonymous "package" of data. SADI accomplishes this by distinguishing various data or service control elements by their ontological type, as described below.

#### Input data is "decorated" until it becomes an instance of the Output Class

**Figure 2:**
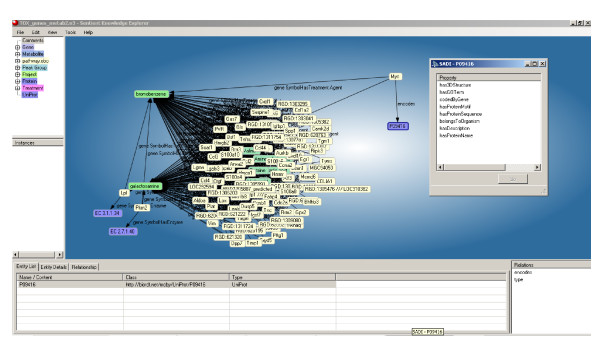
**The SADI Plug-in to the IO Informatics Knowledge Explorer**. In this image, we have selected a node on the canvas representing UniProt protein P09416 and a right-click has raised the SADI Plug-in menu. The menu is derived by requesting the rdf:type information for the selected node, and then searching the SADI registry for all Semantic Web Services that consume that data class. From the discovered services, the RDF predicates that are created by those services are then displayed in the menu for the user to select. Clicking "GO" invokes the selected services and the returned data is added to the graph on the canvas.

**Figure 3:**
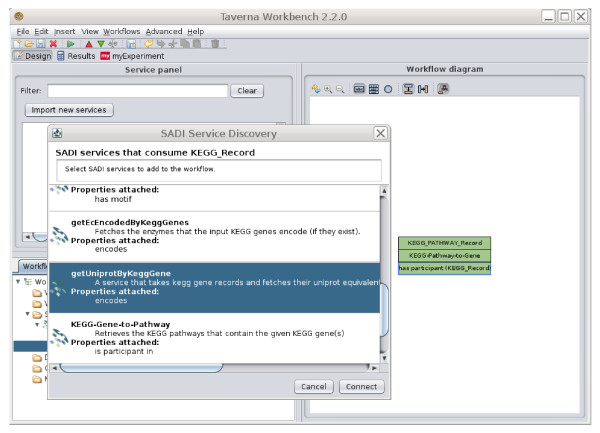
**The SADI Taverna Plug-in**. In this image, the user has already placed the SADI service "KEGG-Pathway-to-Gene" on the canvas. This service reports that it consumes data of type "KEGG_PATHWAY_Record" (upper/input port) and attaches the predicate "has participant" with a value of "KEGG_Record" - the participants in this KEGG pathway (lower/output port). The user has now right-clicked on the output port of this service to obtain the SADI Plug-in window. SADI has semantically examined the properties of the output from the KEGG-Pathway-to-Gene service and has discovered services capable of operating on those properties. Among these is a service "getUniprotByKeggGene" (selected and highlighted in blue) which will provide the "encodes" annotation on any genes that appear in that service output. To add the service, the user simply clicks the "Connect" button, and the services will be automatically, and accurately, pipelined together with no additional manual intervention required.

This is the critical aspect of the SADI specification that leads to SADI's striking interoperable behaviours; moreover, this manner of modeling services also provides simple solutions to problems that would otherwise require project-specific standards (e.g. the mapping of input to output in a multiplexed invocation, as described later). Simply put, after a service analyses the predicate/values attached to a given input node, it then adds the analytical output to that same node through one or more new predicate/values. The output is associated to the input as a new property of that input URI (compare the URI of the main node "person:1" in both Figure 1C and Figure 1D). All of the predicates and values added by a service are defined in the Output OWL Class, and as such, output data is then rdf:type'd according to that output Class definition. More importantly, appropriate services can be discovered based on the properties they add. For example, Figure 2 shows the SADI Plug-in to the IO Informatics Knowledge Explorer[8]. The UniProt protein P09416 has been selected, and in the panel to the right, the SADI plug-in is displaying all of the properties of that protein (e.g. 3D Structure, GO Annotations, etc.) that are available through invocation of one or more SADI services. Similarly, Figure 3 shows the plug-in to Taverna, where a similar menu of property/values is provided based on the data-type that will emerge from the output port of the currently selected service on the canvas.

Given the rapidly increasing size of bioinformatics datasets, and the movement to cloud-based computing, SADI natively supports the ability to pass data by reference. In the case of both input and output data, the URI of the owl:Individual may be annotated with an rdfs:isDefinedBy predicate. The Object URI of that predicate, when resolved, should provide triples containing any missing data for that Individual. As such, it is possible to pass large data objects from service-to-service without necessarily passing the data, but still provide the ability to retrieve that data in a standards-compliant way.

### Concrete Examples of SADI Service Description and Invocation Messages

**Figure 4:**
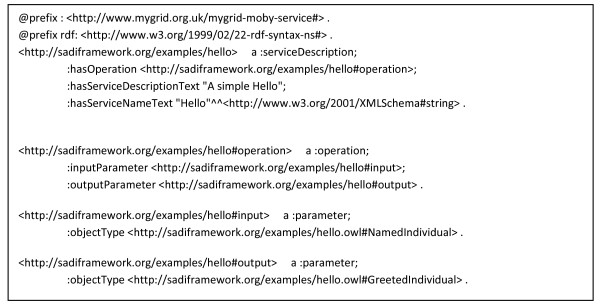
**An SADI service description in N3 format (for readability)**. The document describes an instance of the serviceDescription class from the mygrid-moby-service ontology. In this "Hello" example, there is a single operation (all SADI services consist of a single operation), with a single input parameter that is of type NamedIndividual from the "hello.owl" ontology, and a single output parameter of type "GreetedIndividual" from the same ontology. This document can be retrieved (in RDF/XML format) by calling HTTP GET on the service's endpoint at http://sadiframework.org/examples/hello

**Figure 5:**
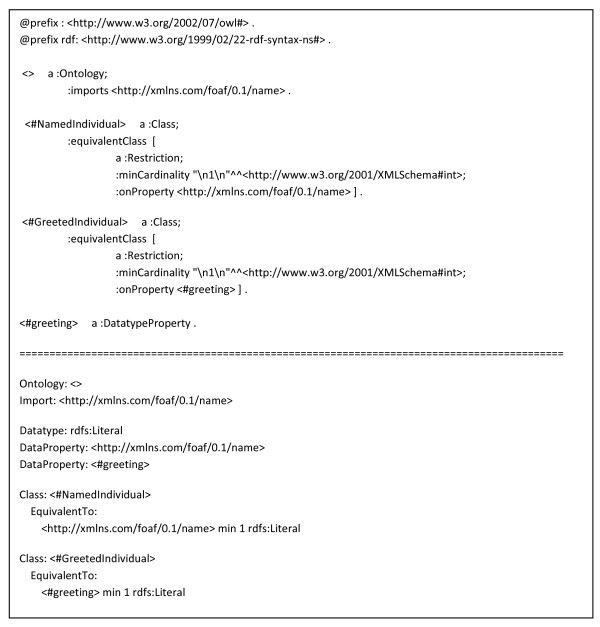
**The OWL Ontology, shown in both N3 format (above the divider) and in Manchester OWL syntax (below the divider), describing the "Hello" service's input and output classes**. The NamedIndividual (input) class declares that the service consumes any URIs that include at least predicate of type "name", from the FOAF ontology. The GreetedIndividual (output) class indicates that the SADI service will add the "greeting" property to the input data, and that "greeting" is a Datatype Property.

**Figure 6:**
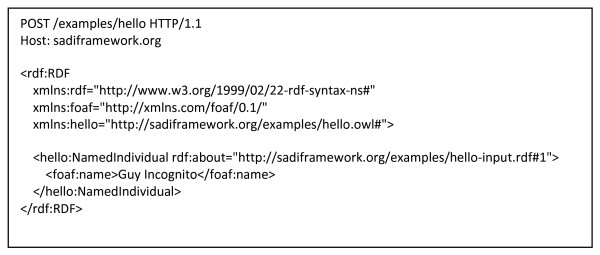
**Invocation Message**. This is the full HTTP message sent to invoke the "hello world" service. It utilizes the HTTP POST method, and is sent to the service endpoint at http://sadiframework.org/examples/hello. The message body consists of an RDF/XML instance of the NamedIndividual class (as per the Hello service's hello.owl ontology), with the property "name" and a value of "Guy Incognito". The URI of this individual is http://sadiframework.org/examples/hello-input.rdf#1.

**Figure 7:**
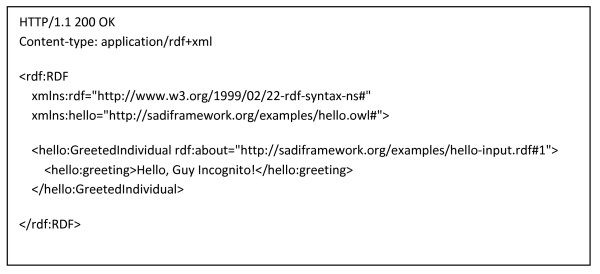
**Synchronous Response Message**. This is the full HTTP message sent in response to the invocation message from Figure 3. It is an RDF/XML instance of the Hello service's output class - GreetedIndividual. As per that class definition, the instance carries a "greeting" predicate, with the value "Hello, Guy Incognito!". Note that the URI of the GreetedIndividual is identical to the URI of the NamedIndividual input, as per the SADI best-practices.

**Figure 8:**
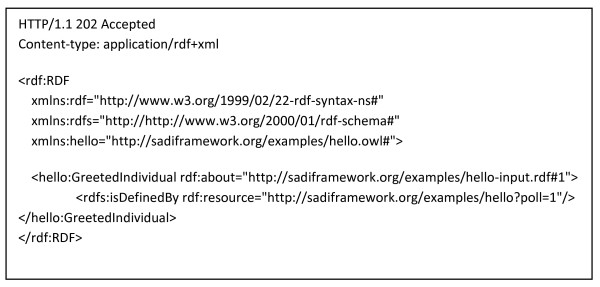
**Asynchronous Response Message**. This is the full HTTP message sent in response to the invocation message from Figure 3, as it would appear if the Hello service were implemented asynchronously. It is an RDF/XML instance of the Hello service's output class - GreetedIndividual, but unlike the response message in Figure 4, the output data is not yet attached. Rather, the input URI is now decorated with the "isDefinedBy" predicate from the RDF-Schema standard vocabulary. The value of that predicate is a URL which can be polled by the client until the data is ready. The response message carries the HTTP Header standard response code of 202 "Accepted but incomplete".

**Figure 9:**
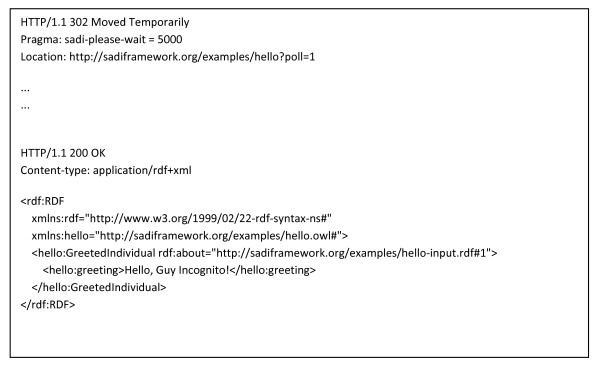
**Asynchronous Polling Response Messages**. At the top of the figure is the response obtained when polling for an asynchronous response when the data is not yet ready. The "redirect" (HTTP 302) header is used to indicate that the client should call a URL (in this case, the same URL). The ellipsis indicate repeated polls of the same URL. When the data is ready, the full response is sent with an HTTP 200 header. Note that the response message is now identical to that of a synchronous service (Figure 4).

Figures 4, 5, 6, 7, 8 and 9 provide concrete examples of the guidelines described above, in the context of a "Hello World" style example. Figure 4 shows the service's description, which is obtained by performing an HTTP GET on the service's endpoint. This document contains both the human-readable annotations of the service, as well as the machine-readable pointers to the service's input and output OWL Class definitions. Figure 5 shows the ontology describing the input and output OWL Classes. The input Class is composed of a single property restriction indicating that any incoming data must have at least one "name" predicate. In this way, SADI allows data to be re-classified as valid input to a service, even if it had been generated from another ontological framework, so long as it carries the required properties. This provides extreme flexibility in data-to-service matchmaking. The output OWL Class is similarly composed of a single property restriction indicating that the output from the service will include the "greeting" predicate. It is this "greeting" property that is indexed by the prototype SADI registry, and can be used for service discovery. Put another way, the *function *of this service is to generate the "greeting" property of an input URI based on its "name" property. This equivalency between a Web service's function and the creation of novel properties, to our knowledge, completely unique to the SADI Web service model, and is largely responsible for the semantic behaviours that will be demonstrated in the Utility section of this manuscript. Figure 6 shows a complete input message, passed by HTTP POST to the service's endpoint. As described earlier, the message has no additional scaffold or messaging format. It is simply an RDF individual corresponding to the service's input OWL class. Similarly, Figure 7 shows a complete output message from the same invocation of the Hello service. Once again, it is nothing more than an RDF individual of the service's output OWL class, but importantly, the URI of that individual has not changed. In this way, it is trivial to determine which output was derived from which input when multiplexing service invocations.

## Complex services

### Multiplexing service calls

The use of RDF, and lack of message scaffolding makes multi-plexing service invocations trivial, and is an important feature that distinguishes SADI from most prior Web service and Semantic Web service frameworks. Any given service invocation RDF document may contain one or more instances of the input class, and in this manner, multiple service invocations can be "bundled" into a single POST. This allows the service provider to optimize the way that request is managed, for example, by distributing it over a computing "farm". Because the URI of the input instance(s) is preserved in the output instance(s), no additional mark-up, and no new standards, are required to determine which output maps to which input. From the service provider's perspective, this means that no effort is required to re-compile the output message, since it is simply a concatenation of all outputs from all compute runs. From a client perspective, it means that no SADI-specific software is required to invoke a SADI service, even when multiplexing thousands of inputs.

### Asynchronous services

In keeping with our longstanding recognition of the importance of asynchronous service invocation within the BioMoby project, support for asynchronous services was a high priority in the design of SADI. For long-running services, SADI proposes a very lightweight, pure HTTP approach to asynchronous invocations. In an asynchronous service, input URIs are decorated by the predicate 'rdfs:isDefinedBy' with a temporary, service-specific URI as its value, and are immediately returned to the client. In compliance with the defined usage of this predicate [17], the interpretation of this statement is that the input URI is further defined by resolving the temporary URI provided by the service. This incomplete output data is contained in the body of an HTTP 202 ("Accepted but incomplete" [18]) response message (see Figure 8), in accordance with the proper usage of the HTTP 202 header. The service-specific URIs, when resolved by GET, either return the output graphs (if the service operations are complete) or the "redirect" (HTTP 302) header is used to indicate that the client should re-call a URL (in this case, the same URL; see Figure 9). Since this is the standard behaviour of most HTTP client programs, this helps ensure that most existing Web-enabled software will deal appropriately with Asynchronous SADI services without the need to invent a novel standard. To assist clients in regulating their repeat requests on an asynchronous service, we currently pass a HTTP Retry-After directive in the response message header. In future implementations, a Web services Resource Framework [19] reference may be passed in the HTTP 202/302 headers, providing information about the state of the asynchronous service, and to assist clients in determining when an output graph will be available. Supplementary information showing more complex sample message structures is provided at [20].

### Services with control-parameters

Since SADI services are invoked by a non-parameterized POST, all information required by the service to define its behaviour must be contained within the invocation message. For services that have settable parameters (for example, selection of a BLOSUM matrix and/or e-value cutoff in BLAST), such information is passed to the SADI service as an independent RDF graph within the same invocation message. The service provider specifies an OWL Class in which the parameters and value-restrictions for their interface are defined. In the myGrid-Moby Ontology, these are differentiated from "data" input Classes by virtue of being attached to mygrid:secondaryParameter nodes in the service definition RDF document. When invoking a service, client software simply creates an instance of this secondaryParameter Class, and passes it to the service along with the Input data instances. The service then extracts the URI that is rdf:type [TheirParameterClassname] and collects the parameter information from this object to configure the service prior to analysing the data. Again, no project-specific standards or message structures are defined by SADI to achieve this goal - parameter data is simply RDF data placed into the input message, and typed according to the Class-name provided by the service host.

## Utility

Observing the behaviors of several implementations of SADI client software will help demonstrate both its utility, as well as how many common problems with Web service interoperability are effectively resolved by this approach. In the first example, we will demonstrate how SADI can be used to simplify the interaction between an untrained end-user and the myriad resources they may need to dynamically access. The second example will show how SADI contributes to the Linked Data movement by dynamically generating Linked Data triples that can be queried, and also demonstrates the simplicity with which SADI-compliant Web services can be pipelined together.

### Example 1 - the SADI Plug-in to Taverna

Taverna is an open-source workflow design and enactment workbench that allows users to "drag-n-drop" Web Services from a menu of available resources onto a canvas, and link them together into an analytical pipeline.

We have created a SADI plug-in to Taverna (described in detail here [7]) that assists users in discovering the service they need and automatically connecting it correctly into the workflow. When an output port of a service is selected in Taverna, the SADI plug-in provides a menu of relationships that can be attached to the type of data that will flow out of that port when the workflow is executed. This list is obtained by querying the SADI registry for services that consume that data-type as input, and the relationships attached by each service are collected and displayed to the user. To add that service to the workflow, the user simply selects their property of interest from the menu. The service is added, and automatically properly connected to the previous service (a process that can be quite difficult in Taverna, depending on the complexity of the service interfaces being connected). For example, if the user has selected a port from which gene identifiers will emerge, the SADI menu might include "encodes Protein" as a property that can be generated by the next service. The inclusion of the semantic relationship ('encodes') between the selected data-type, and the data-type that is going to be generated by the service is (as far as we are aware) unique to SADI, and we believe that this will make the selection of a desired service more intuitive for our target end-users. Given that there are various relationships between genes and proteins (genes are regulated-by proteins, genes encode proteins, etc.) clarity around this relationship is not trivial with respect to selection of an appropriate service by our target end-users.

### Example #2: The SHARE SPARQL query client

A slightly more complex example of usage is presented by our Semantic Health And Research Environment (SHARE) prototype query system [21]. SHARE connects the SADI middleware to the Pellet [22] SPARQL query engine and DL Reasoner. Predicates presented to Pellet from SPARQL queries are "intercepted" and passed to SADI to be used for Web service discovery and automatic invocation. Output data from the invoked services is added into Pellet's local triplestore. In this way, a query-specific triplestore is dynamically generated as a query is being processed; effectively, the database required to answer the question is automatically generated as a result of the question being posed.

This approach has features of many prior attempts at data integration in that (a) it is service oriented, (b) it is similar to link-integration in that every node in the graph is a resolvable URL, (c) it offers the "data freshness" of view-integration since data is being dynamically discovered (or generated) by the source, and (d) it offers the reproducibility of a warehouse, since the graph that results from a SHARE query can be permanently stored and explored using a variety of tools.

## Discussion

### Justification for creating a new Semantic Web service standard

A decade ago, Stein expressed concern that, because a wide array of different approaches to Web service provision were emerging "a chaotic world of incompatible bioinformatics data standards will be replaced by a chaotic world of incompatible web service standards" [23]. It would be difficult to argue that those words were not prophetic! In an attempt to enhance interoperability between these resources *post facto*, independent projects began using semantics to help map between the data elements and representations used by each resource. These "Semantic Web service" initiatives themselves, however, took various approaches in their utilization of semantics.

Preceding both Semantic Web technologies and the widespread emergence of Web services in bioinformatics, TAMBIS [24] was a mediator system in which wrappers containing resource-specific queries were mapped to an overarching ontology of bioinformatics concepts. Thus the semantics of TAMBIS is separate from the individual resource interfaces, and the semantic layer acts to re-write multi-concept queries such that individual components of that query are executed by one or more resource-specific wrappers.

*my*Grid [25] used an extensive bioinformatics domain ontology to annotate traditional bioinformatics Web services within a formal model called "Feta" [14], designed primarily to enhance service discovery, rather than automate multi-service composition. Feta, thus, adds semantics to traditional Web services at the level of its own annotation of a service interface.

OWL-S [12] seeks to improve Web service interoperability by providing a standard OWL ontology for the description of Web services. OWL-S goes beyond the capabilities of WSDL in the sense that it aims to describe the effects of web services on the real world (e.g. adding a charge to a credit card). OWL- S describes the actions of a Web service in a similar manner to how the actions of an agent are described in the planning domain of AI. Each service has a set of pre-conditions and post-conditions which are expressed as boolean formulas over a set of state variables. OWL-S is complex and is under ongoing development.

SAWSDL (Semantic Annotations for WSDL)[13] is an extension to WSDL that attempts to bridge the gap between the world of syntactically described Web services and semantically described Web services. SAWSDL allows a service provider to "tag" parts of a WSDL service description with semantic annotations. These annotations either specify how to translate an XML schema element to/from an ontology instance in another language such as RDF (via the liftingSchemaMapping and loweringSchemaMapping attributes), and indicate that an XML element corresponds to a certain class in an ontology (via the modelReference attribute).

WSMO (Web service Modeling Ontology) [26] is a research project that has the same general goals as OWL-S. In contrast to OWL-S, WSMO uses its own modeling language, WSML (Web service Modeling Language) for encoding Web service descriptions. One advantage of WSML over OWL-S is that it has built-in syntax for encoding the boolean formulas that are used to describe the pre-conditions and post-conditions of the services. In contrast, OWL-S employs a more *ad hoc *approach where the formulas are encoded as XML literals or string literals in an external syntax such as PDDL [27].

caBIO (part of caCORE [28]) designed a traditional Web service API describing all "valid" operations for a given set of biological objects. Within the XML sent-to or received-from caBIO services are semantic annotations compliant with a (vast) domain vocabulary. Thus the semantics of caBIO data are contained in the values of XML elements, and the "meaning" of those XML elements themselves are defined by the caBIO API.

BioMoby [11] carries its semantics in the data-structures themselves, and unlike caBIO, does not constrain what operations can be done on any given biological object. BioMoby requires service providers to utilize a common, end-user-extensible ontology of biological data-types, and to consume and produce XML serializations of instances of that ontology. The BioMoby ontology is both hierarchical, and partitive, thus the element name at any given position in the resulting XML serialization, and its child-element structure, can change without changing the semantics of the data. This enhances interoperability because (a) the semantics of the data are self-describing and embedded in the data, and (b) complex messages can be utilized by more simplistic services by simply paying attention to those data-components that they understood. As a result, assembly of BioMoby Web services can be fully automated since the "meaning" of any given data message can be reliably interpreted by the recipient without the need of mediators. Unfortunately, this flexibility in the XML representation of the data precludes the ability to use XML Schema to describe the syntax of the message, and thus traditional Web service tools are of limited utility. Moreover, BioMoby's XML serialization is non-standard and only understood by other BioMoby services, hampering interoperability outside of the project.

SSWAP [29] also carries the semantics of the data in the message itself, however it utilizes Semantic Web standards to do so. SSWAP defines a shared, lightweight OWL model of a service interface, where RDF-XML instances of this model are used as both the interface definition and as the "container" of the input and output data during service invocation. Because OWL-RDF cannot (reliably) be described in XML Schema, and because SSWAP includes the service interface model as part of its required messaging "scaffold", SSWAP is also incompatible with traditional Web services toolkits, and requires project-specific tooling, but exhibits significant interoperability and automatability with other SSWAP services.

Though some of these approaches might still be considered "emergent", even the more mature ones are not in widespread use outside of their own communities. Moreover, each approach attempted to inject semantics at a different position within the normal Web Services paradigm, making many of these Semantic Web service approaches incompatible with one another.

To justify our creation of (yet) another approach to Semantic Web service provision, we must discuss both published and subjective observations of Web service functionality, and pinpoint areas that continue to be problematic with respect to either service discoverability, or service interoperability. Clearly, if we cannot demonstrate the potential for a significant improvement over the *status quo*, service providers will have no motivation to adopt this approach, and the project will fail. Here, then, are the core observations that compel us to attempt a novel strategy.

First, we, and others [14,30], noted that Web services in bioinformatics (and other scientific domains) exhibit only a small subset of the full range of complex behaviours that service-oriented Architectures allow. With few exceptions, bioinformatics Web services are independent, idempotent, stateless, transformative, and atomic. This stands in stark contrast to Web service solutions to, for example, the ticket-ordering use-case that is commonly discussed in this domain. Almost invariably, bioinformatics Web services consume a specific input data type, and in a stateless and atomic operation, return related output data type(s) generated by whatever transformation the service executes on that input. That most services are transformative in this way suggests that attempting to declare or model the underlying business-process may be unnecessary in the bioinformatics domain - to quote Goble again, "any integration technology should only be as heavy as it needs to be". Indeed, this observation was made by both the Feta and BioMoby projects [11,14], though both Feta and BioMoby acknowledged the need for some level of simple service *type *annotation to assist in discovery.

A second important consequence of the observation that bioinformatics services are transformative has not (to our knowledge) been previously highlighted; that is that the transformation of input to output implies that there is some *relationship *between that input and output, and this important metadata is not being captured or utilized by any current framework. We believe that these relationships, while not capturing the service's "business process" *per se*, capture with great accuracy the *purpose *of the service; moreover, through observations made on the students of training courses in Web service workflow composition, we (subjectively) concluded that these relationships are likely a more accurate reflection of the way our end-users think about these data transformations, versus annotating the algorithmic function as is done in BioMoby and Feta. For example, biologists do not execute a BLAST analysis because they wish to run a sequence similarity matrix over their input data; they execute a BLAST analysis because they are interested in finding sequences that are homologous to their input sequence - they are interested in the homology relationship, not the BLAST algorithm. As such, we believe that capturing these entity-relationships as service annotations is an important criterion for enhancing discovery of relevant services by our target users. This observation lead to our second core best-practice: that services add their output to the input node via a meaningful property describing the relationship between input and output, and services may therefore be indexed and discovered based on that property.

Our third observation was twofold. On one hand, we noticed a general sense of disdain, bordering on frustration, within much of the bioinformatics community with respect to the SOAP protocol in general, and the incompatibilities between various language and platform-specific implementations of SOAP. With the distinct exception of the National Cancer Institute's caBIO framework, bioinformatics resources only rarely implement SOAP interfaces that utilize the Object-oriented style that SOAP allows, and even fewer take advantage of the rich features of the SOAP envelope such as intermediaries and message paths. Other than caBIO, almost all bioinformatics Web interfaces are straightforward, single-operation request/response. For example, the SOAP interface of TogoWS [31] provides a KeggGetEnzymesByPathway function that consumes a KEGG pathway identifier and responds with a list of related Enzymes. For these kinds of services, the overhead of SOAP is (demonstrably) unnecessary, so we feel it would be preferable to avoid SOAP entirely. On the other hand, there is an increasingly positive attitude in our community towards "RESTful" architectures [32]. It is worth taking a moment to dissect this goodwill, however, since it is in our opinion slightly misplaced. Few, if any, bioinformatics interfaces that claim to be RESTful are *truly *following a REST architecture. To be RESTful, all entities would be named resources whose states are manipulated through a limited number of methods. This is not a trivial architecture to achieve in practice, and most importantly is not, *in any way*, the same as declaring that all parameters for all functions should be part of a URL. Such interfaces (i.e. the vast majority of "RESTful" interfaces in bioinformatics) would better be described as CGI GET-based interfaces. For example, the "REST" interface of PhyloWS [33] consumes a specially-formatted query URL including a clade identifier and other key/value parameters, and returns a phylogenetic subtree. There is no identifiable resource whose state is being manipulated by that operation, and while it might be argued that every conceivable query is its own GET-able resource, such an argument would be a contrived interpretation of REST philosophy. As such, we believe that the bioinformatics community's goodwill is directed at interfaces that limit themselves to "pure" HTTP Protocol, rather than REST *per se*. As such, we decided to utilize straightforward HTTP GET and POST for SADI, relying heavily on standard HTTP response codes for special cases, though we do not claim SADI to be "RESTful".

Fourth, after observing the barriers to up-take of both BioMoby and SSWAP, it became clear that project- or protocol-specific message scaffolding should be avoided. As such, the SADI recommendation is to pass data only, with no scaffolding whatsoever.

Finally, we made a subjective evaluation of the cause of failure in (most) precedent interoperability architectures, and concluded that, in our opinion, XML Schema *is the problem *and should be abandoned. To briefly justify this conclusion, we observe the following: XML Schema has been described as "far and away the most complex data model ever proposed" and "seriously flawed" [34]. Bring into this complexity the number of different aspects of our target domain that need to be represented (Strömbäck et al. found 85 different schemas within the sub-domain of systems biology alone[35]), and there is immediately a requirement for either schema standardization, or schema mapping to facilitate interoperability. Schema standardization is "prohibitively time-consuming" [36], and though there have been numerous attempts to automate schema mapping - that is, the ability for two schema to exchange data, as would be required to automate the interaction between arbitrary Web services - none have proven reliable in an open-Web situation [37]. Automated Schema mapping is likely an AI-complete problem since it requires the mapping of arbitrarily chosen natural-language labels (XML tags) to one another based on the semantics of either the tag or its child-content. As such, Schema mapping approaches are unlikely to yield an acceptable result in the foreseeable future. This barrier has had significant and destructive consequences beyond the obvious thwarting of interoperability. The inability to automatically map between Schema has resulted, counter-intuitively, in an *increase *in the complexity of Web service interfaces. Since it is extremely difficult to pipeline traditional Web services together reliably, there is little point in making their operations highly granular; it is more "efficient" to simply execute the entire service operation as a single function-call. This, in turn, increases the complexity of the input and output messages[38] making schema mapping even more difficult. Our final observation is that, there is considerable early-adoption of Semantic Web technologies in the life sciences, with several significant organizations already publishing their data in RDF format (e.g. UniProt [39]). If we continue using XML Schema-based services, we may soon find ourselves mapping semantically rich data back into semantically impoverished XML in order to analyse it (this is, in fact, the purpose of the SAWSDL specification!). This would defeat the purpose of utilizing Semantic Web technologies in the first place. Clearly, more is gained by natively taking advantage of the enhanced interoperability inherent in RDF representations of data, than is gained through trying to support legacy Schema-based interfaces. For all of these reasons, we utilize RDF/OWL as both our interface description and messaging layer, and require it for all SADI-compliant interfaces. Moreover, we suggest that our community's continued adherence to traditional Schema-based Web service specifications will, at best, be destructive to their attempts to be interoperable. To quote Lincoln Stein, "to achieve seamless interoperability among online databases, data providers must change their ways" [23].

### SADI and the Linked Data movement

The behaviour of SADI is consistent with, and in fact furthers the goals of the Linked Data[40] community. Consider, for example, what happens in a SADI service workflow, such as those automatically generated by the SHARE client. Input data is passed to a service, and comes back with output data attached. That output data may be utilized as input to a subsequent service, and so on. As the data flows through that workflow, a rich Linked Data graph is being constructed where every input is semantically linked to every associated output. This graph of dynamically generated data can be integrated with traditional static Linked Data resources, and queried or explored using standard Linked Data toolkits.

### SADI and the Semantic Web

SADI merges the domains of Web services and the Semantic Web in a novel way. Every service generates one or more "edges" on an RDF graph, where the edge that will be generated is defined as a property restriction in an OWL ontology. Therefore, in SADI, OWL property restrictions "represent" potential services, and therefore SADI can be used to generate instances of OWL classes through service discovery based on these property restrictions. OWL, effectively, becomes an abstract workflow language. Moreover, any OWL document - whether created for this purpose or not - can be used by SADI-enabled software to retrieve instance data, so long as SADI services exist that map to the properties used in the ontology. Thus SADI is able to take advantage of any Semantic Web ontology.

Finally, while the bioinformatics community continues to utilize large, complex, semantically opaque flat-files, we believe that SADI (and the Semantic Web in general) starts to provide greater impetus to break-out the semantics of these files and increase the granularity of both data and services in the bioinformatics space. While SADI does not dictate the nature of the input and output data, it would be somewhat absurd for a SADI BLAST service to output a BLAST flat-file linked to its input sequence by a (nonsensical) "hasBLASTReport" property. Instead, the Linked-Data Web that SADI services build make it much more useful to output a parsed BLAST report, where each "hit" is linked to the original input sequence through some form of "sharesSimilarityTo" relationship. Thus, by challenging service providers to make their services discoverable through a biological relationship, rather than a algorithmic one, we believe SADI will provide the incentive to move beyond semantically opaque text reports and start explicitly encoding the semantics contained in those documents, resulting in a much richer data ecosystem.

### SADI and other emergent Semantic Web service standards

As noted above, several of the existing Semantic Web service approaches are relatively new, and may still experience widespread adoption. Among these, the SAWSDL specification seems to be gaining considerable traction, though for reasons discussed earlier, we have some concerns about the utility of this standard in an RDF-based world, and about the lack of rigour in the standard itself. Description of SADI services using the SAWSDL standard is trivial, but not particularly useful. SAWSDL enhances traditional WSDL documents by indicating a semantic type for the service's input and output XML elements, and indicates a "lifting" or "lowering" schema to guide the transformation of RDF data into XML and back again. In SADI, the semantic types are simply the OWL Classes that the service provider declare as their input and output. Moreover, because the service natively consumes RDF there is no need for a lifting or lowering schema (or at worst, the lifting and lowering is an identity transformation). Nevertheless, since the SAWSDL specification gives no guidance as to the format of these lifting and lowering schemas, or how to interpret them, and since OWL Individuals cannot reliably be described using XML Schema, there will need to be an additional level of, as yet non-standardized community agreement before SAWSDL services (SADI or otherwise) could expect to be interoperable. Moreover, the myGrid/Moby service ontology contains far more detailed annotation than a SAWSDL document, and these detailed annotations are useful for both service discovery as well as service maintenance and testing. As such, while SADI is superficially compatible with the SAWSDL standard, we find the standard itself lacking for our purposes.

### Limitations of SADI

SADI suffers from the same limitations that pose barriers to other Web service and Semantic Web projects [41]. As an interoperability system, the utility of SADI is entirely dependent on the number of providers who adopt its conventions. We recognize that there is extensive tooling support for traditional Web services and there is a perceived simplicity of XML compared to RDF/OWL. Moreover, there are thousands of legacy bioinformatics Web services that are not interoperable (neither with each other, nor with SADI services), and thus there would appear to be little benefit to becoming an early-adopter of SADI. To counter this, we have created software libraries that partially automate the process of service construction in both Perl and Java. Similar to the "Dashboard" application for BioMoby[42], a plug-in has been created that integrates a SADI service development environment into the Protégé [43] ontology editing application, where the user designs the ontologies describing their data, the plug-in creates the service scaffold, and the provider adds their business logic, setting the values of "stubs" provided by the service scaffold. This automation is possible because the behaviors of SADI services are predictable, and thus the code for SADI services is similarly consistent and predictable. In addition, we believe that the SAWSDL specification, together with XML transformations, will allow us to build semi-automated "wrappers" around traditional Web services that will make them SADI-compliant (at the expense of a loss in semantic richness versus creating a native SADI service). In this way, we hope to bootstrap the SADI project by first simplifying the task of service provision, and then by creating a core set of interoperable services that these Providers can link into. At the time of writing, there are more than 400 bioinformatics and chemoinformatics services available in the SADI registry[44], and several hundred more will be published by our team of collaborators by the end of this year.

The reliance of SADI on the Semantic Web also exposes limitations. In particular, success of the SADI architecture (like the success of the Semantic Web itself) will largely depend on widespread re-use of publicly-available and well-defined ontological predicates, and the definition of service inputs in terms of OWL restrictions on these properties. Unfortunately, the majority of focus in the Semantic Web efforts of the health-care and life science community thus far has been on defining classes, rather than predicates; asserting class-hierarchies without formally defining what properties a member of that class is expected to have, or what distinguishes members of one class from another. We hope, however, that the power we have demonstrated in these prototype implementations provides a sufficiently compelling argument to initiate the evolution of a slightly higher level of Semantic Web complexity in the health-care and life-sciences space.

## Conclusions

SADI proposes a set of conventions and best-practices, within the scope of accepted standards for Web services and the Semantic Web, that enable the creation of bioinformatics software with novel interoperable and integrative behaviors. These were derived by examining the "nature" of Web services in the bioinformatics domain, and observing and subjectively evaluating how these services are found and used by biologists and informaticians. The resulting approach, we believe, accurately models both the services and the end-user requirements for dynamic and automated discovery of relevant services, automated pipelining of these services, and integration of the resulting data.

## Availability and Requirements

SADI is an open-source project and its supporting codebase is hosted at Google Code (http://sadi.googlecode.com). The SHARE demonstration is available for public access (http://biordf.net/cardioSHARE/). The SADI Plug-in to Taverna is available at the SADI homepage (http://sadiframework.org). The SADI Plug-in to the Sentient Knowledge Explorer is not publicly available at this time, but will be released late in 2011.

## List of Abbreviations Used

HTTP: HyperText Transport Protocol; OWL & OWL-DL: Web Ontology Language - Description Logic; RDF: Resource Description Framework; SADI: Semantic Automated Discovery and Integration; SAWSDL: Semantic Annotations of Web service Description Language; SHARE: Semantic Health And Research Environment; WSDL: Web service Description Language; XML: eXtensible Markup Language

## Competing interests

The authors declare that they have no competing interests.

## Authors' contributions

MW conceived of SADI, directed its development and the development of other plug-ins and libraries, and wrote the manuscript. LM implemented the SADI core codebase and supplementary libraries, created the SHARE prototype client, created the Knowledge Explorer plug-in, in collaboration with David Withers created the Taverna plug-in, wrote numerous SADI services, and edited the manuscript. BV optimised the automated workflow pipelining behind the SHARE client, contributed to the core code and libraries, created numerous SADI services, and contributed large portions of the manuscript. All authors read and approved the final manuscript.
